# Parathyroidectomy Improves Restless Leg Syndrome in Patients on Hemodialysis

**DOI:** 10.1371/journal.pone.0155835

**Published:** 2016-05-19

**Authors:** Roberto Sávio Silva Santos, Fernando Morgadinho Santos Coelho, Bruno Caldin da Silva, Fabiana Giorgeti Graciolli, Wagner Velasquez Dominguez, Fabio Luiz de Menezes Montenegro, Vanda Jorgetti, Rosa Maria Affonso Moysés, Rosilene Motta Elias

**Affiliations:** 1 Department of Medicine, Division of Nephrology, Universidade de São Paulo, Brazil; 2 Department of Medicine, Division of Neurology, Universidade Federal de São Paulo, Brazil; 3 Universidade Nove de Julho – UNINOVE, São Paulo, Brazil; 4 Department of Medicine, Head and Neck Surgery Division, Universidade de São Paulo, Brazil; Oasi Institute for Research and Prevention of Mental Retardation, ITALY

## Abstract

**Background:**

Restless leg syndrome (RLS) is a sleep disorder with high prevalence among patients on hemodialysis. It has been postulated that high phosphate and high parathyroid hormone may be implicated in its pathogenesis. Standard international criteria and face-to-face interview are not always applied.

**Methods:**

this was an interventional prospective study in which 19 patients (6 men, aged 48±11 years) with severe hyperparathyroidism were evaluated. RLS diagnosis and rating scale were accessed based on the International RLS Study Group pre- and post-parathyroidectomy. Patients also underwent standard polysomnography.

**Results:**

At baseline, RLS was present in 10 patients (52.6%), and pain was the most reported symptom associated with the diagnosis. Patients with RLS had higher serum phosphate (p = 0.008) that remained independently associated with RLS in a logistic regression model, adjusted for hemoglobin, age and gender (HR = 7.28;CI = 1.14–46.3, p = 0.035). After parathyroidectomy, there was a reduction of serum parathyroid hormone, phosphate, calcium and alkaline phosphatase, and an increase of 25(OH)-vitamin D, and Fetuin-A. Parathyroidectomy alleviated RLS (from 52% to 21%; p = 0.04), which was accompanied by a decrease in severity scale, in association with relief of pain and pruritus. Polysomnography in these patients showed an improvement of sleep parameters as measured by sleep efficiency, sleep latency and percentage of REM sleep.

**Conclusion:**

RLS is associated with high levels of phosphate in patients with severe secondary hyperparathyroidism on hemodialysis. Pain is most reported complain in these patients. Parathyroidectomy provided an opportunity to relief RLS. Whether the reduction of serum phosphorus or parathyroid hormone contributed to this improvement merits further investigation.

## Introduction

Restless leg syndrome (RLS) is a movement sleep disorder characterized by voluntary leg movements prompted by an urge to move, which is often associated with unpleasant paresthesia. The prevalence of RLS in the general population is approximately 5 to 10% of adults [1,2], while can reach prevalence as high as 70% [3] [3–6] among patients with end-stage renal disease (ESRD). The impact of RLS on cardiovascular events and mortality is high for both general population [7] and patients with ESRD [3,8]. Nonetheless, its underlying pathophysiology is not completely elucidated.

Elevated levels of serum phosphate [9] and parathyroid hormone (PTH) [10] were already associated with the pathogenesis of RLS in patients with ESRD. A recent meta-analysis found, however, no association between PTH and RLS, evaluating patients on dialysis as an entire group, without distinguish those with and without hyperparathyroidism [11]. Secondary hyperparathyroidism, a condition relatively common in ESRD patients, seems to be the ideal scenario for RLS, as usually exhibit high phosphate and PTH. Parathyroidectomy, which is a surgical treatment of this condition, on the other hand, seems to be the ideal intervention to confirm the association between phosphate, PTH and RLS, as it promptly corrects these biochemical abnormalities.

So far, studies on RLS in secondary hyperparathyroidism are observational, have applied non-validated questionnaires, have not used face-to-face interview to confirm the diagnosis, and have not had focus specifically in RLS. Nevertheless, these studies have observed an improvement of RLS symptoms post-parathyroidectomy, in association with a reduction of phosphate, and an increase of the referred sleep duration.

As objective data obtained from polysomnography is lacking, and the international criteria indicated for the diagnosis of RLS was not usually applied, those results are still debatable. Here, we aimed to verify if there is an improvement of RLS in ESRD on hemodialysis with severe hyperparathyroidism, in a prospective design, pre- and post-parathyroidectomy. In addition, clinical presentation and polysomnography data were also obtained.

## Materials and Methods

### Subjects

Patients were recruited from the chronic kidney disease-mineral and bone disorder (CKD-MBD) outpatient clinic, in a tertiary care hospital in São Paulo, Brazil (Hospital das Clínicas da Faculdade de Medicina da Universidade de São Paulo), from March 1, 2013 to February 31, 2014. Consecutive patients attended during the study period, with a pre-established date for PTX, were invited to participate, regardless of sleep symptoms. Inclusion criteria were patients with ESRD at least 18 years of age undergoing conventional hemodialysis thrice a week for at least 12 months, with severe HPTS and indication for surgery treatment. All patients underwent total PTX with a forearm parathyroid auto graft or subtotal PTX.

We excluded diabetic patients and anyone who was been treated for obstructive sleep apnea, polyneuropathy or RLS. Prior to polysomnography (PSG), demographic characteristics, medical history, laboratory data and prescribed medications were recorded. Pain and pruritus were evaluated using the visual numeric scale.

### Protocol

This clinical study was in conformity with the principles outlined by the Declaration of Helsinki. The protocol was approved by the Research Ethics Boards of the Universidade de São Paulo (Comissão de ética para análise de projetos de pesquisa—Cappesq #0810/11), and all subjects provided written informed consent before participation. We prospectively studied a selected sample of patients with ESRD and severe hyperparathyroidism, on hemodialysis. Patients were invited to undergo clinical evaluation, PSG, and blood work within 0–4 weeks pre- and 12–16 weeks post-PTX.

### Parathyroidectomy (PTX)

Patients selected for PTX were entered in a waiting list according to the chronological order. As our Institution is a tertiary academic Hospital, patients on PTX waiting list were referred from many other dialysis facilities in Sao Paulo province. Even though the original criterion for surgery was time on the waiting list, the severity of symptoms (such as worsening of pain, tendon rupture, new fractures) was actually a main guide to anticipate surgery.

### Laboratory Data

All laboratory analyses were performed by standard techniques, specifically: ionic calcium [reference range (RR) = 4.6 to 5.3 mg/dl; selective ion method]; serum phosphate (RR = 2.7 to 4.5 mg/dl; enzymatic colorimetric method); iron (RR = 59 to 158 μg/dL; enzymatic colorimetric method); ferritin (RR = 30 to 400 ng/mL; eletroquimioluminescence method); transferrin saturation index (RR = 20 to 40%); alkaline phosphatase (RR = 32 to 122 U/L); parathyroid hormone (RR = 11 to 62 pg/ml, immunoradiometric assay); 25(OH)-vitamin D (RR = 30 to 100 ng/mL; quimioluminescence method); serum Fetuin-A (RR = 0.244 to 1,000 ng/mL; Luminex xMAP, Milliplex Analytes, Millipore Corp, St. Charles, MI). All samples were collected in fasting state.

### Subjective daytime sleepiness

The Epworth Sleepiness Scale (ESS) assessed subjective daytime sleepiness. The ESS is a self-administered questionnaire designed to measure the general level of daytime sleepiness. Patients rate on a scale of 0–3 how likely they are to fall asleep in eight different situations commonly encountered in daily life. Total ESS score ranges from 0 to 24; higher scores indicate more subjective sleepiness [12].

### Restless Leg Syndrome evaluation

The diagnosis of RLS was established according to the International Restless Leg Syndrome Study Group (IRLSSG) [13] in a face-to-face interview, by the same observer. Essential diagnostic criteria for RLS included urge to move the legs, worse during rest or inactivity, worse in the evening or night and relieved by movement such as walking. To consider the diagnosis of RLS, patient should answer “yes” to all 4 questions. Pruritus or pain as isolated symptoms, without circadian characteristic and without relief with movement were not considered as RLS. Patients with diagnosis of RLS were asked to fill the international RLS Severity Scale (IRLS) questionnaire. The IRLS has been developed by the IRLSSG and comprises a 10-item scale to enable a well-validated and easily administered measurement of RLS severity. Patients are classified as having mild, moderate, severe and very severe RLS according to scores 1–10, 11–20, 21–30 and 31–40, respectively [14].

### Polysomnography

Full-night assisted PSG was performed to assess all sleep parameters, focusing in periodic leg movements (PLM). All subjects underwent overnight PSG in the Sleep Laboratory of Otorhinolaryngology Division of Hospital das Clínicas, which was performed using a digital system (EMBLA^®^S4500, Embla Systems Inc., Broomfield, CO, USA) during their usual sleep time. The following physiological tests were performed: electroencephalography, electrooculography, electromyography, electrocardiography, and airflow detection by a thermocouple and by nasal pressure. In addition, the following physiological parameters were evaluated: respiratory effort using thoracic and abdominal x-trace belts, snoring and body position by EMBLA^®^ sensors, and arterial oxygen saturation (SaO_2_) and pulse rate by an EMBLA^®^ oximeter. Signals were recorded on a computerized sleep recording system (RemLogicT PSG Software) and seen by a neurologist and sleep specialist blind to the moment of study (pre or post PTX) according standard criteria.

The EEG and leg movements were classified with the use of standard techniques and scoring criteria for sleep stages and arousals from sleep [15]. Obstructive apnea was defined as a >90% reduction of tidal volume for ≥10 seconds in the presence of out-of-phase thoracoabdominal motion, and obstructive hypopnea was defined as a 50% to 90% reduction in tidal volume from baseline for ≥10 seconds with out-of-phase thoracoabdominal motion or airflow limitation on nasal pressure. Apneas were classified as central in the absence of thoracoabdominal motion, and hypopneas were classified as central in the presence of in-phase thoracoabdominal motion and without airflow limitation on nasal pressure. The severity of sleep apnea was classified according to AHI: mild (AHI between 5 and 15), moderate (AHI between 15 and 30) and severe (AHI >30).

### Statistical analysis

Comparisons of continuous values from pre to post-PTX were done by paired t test or Wilcoxon matched test, appropriately. For categorical variables, a chi-square test was performed. Relationships between single variables were examined by the Spearman or Pearson correlation coefficient according to data distribution. Logistic regression analysis was also undertaken with RLS as the dependent variable and with age, hemoglobin and gender as the independent variables. Data are presented as mean ± standard deviation (SD) unless indicated otherwise. A p value <0.05 was considered significant. Analyses were performed with the use of SPSS 21.0.1 (SPSS Inc., Chicago, IL, USA) and GraphPad Prism 6 software (GraphPad Software Inc., San Diego, CA, USA).

## Results

In total, 30 patients were screened of whom 5 did not agree to participate, 2 were excluded because of insufficient sleep at sleep laboratory (sleep efficiency <20%), and four underwent pre- but refused to perform post-PTX PSG. Demographic, clinical and biochemical characteristics pre- and post-PTX of the remaining 19 patients (6 males) who underwent both pre- and post-PTX PSG are described. In general, our study population was relatively young, 48 ± 11 years, with long dialysis vintage [94 (60,131), ranging for 20 to 192 months]. The etiology of ESRD was hypertension in 6 patients (31.6%), chronic glomerulonephritis in 2 patients (10.5%), chronic pyelonephritis in other 2 patients (10.5%), three patients with other causes (15.8%) and 6 patients with unknown cause (31.6%). Three patients (15.8%) informed that they had smoking habits. Five patients were taking vitamin D analogues (2 with and 3 without RLS). None of them were receiving cinacalcet.

RLS was found in 10 patients (52.6%) pre PTX. Pain was the most reported symptom associated with the diagnosis, presented in 6 patients, while paresthesia was reported only in 4 patients. Table 1 shows demographic, clinical, biochemical and sleep parameters characteristics regarding RLS diagnosis. There were no significant differences in most of the baseline characteristics among patients with and without RLS, with the exception of most severe pain and higher serum phosphate among those with RLS. There were no differences in any sleep parameter, with a trend towards higher PLMI in patients with RLS. Logistic regression showed that serum phosphate remained the only independent variable associated with RLS (HR = 7.28; CI = 1.14–46.3, p = 0.035), in a model adjusted for hemoglobin, age and gender. The severity of RLS pre-PTX correlated with the dose of erythropoietin/kg/week (r = 0.689, p = 0.039). There was no correlation between the severity of RLS and serum 25(OH)-vitamin D (p = 0.656).

**Table 1:** Clinical and demographic characteristics stratified by the presence or absence of restless leg syndrome (RLS).

Variable	Absence of RLS N = 9	Presence of RLS N = 10	p
Age (yr.)	48 ± 12	47 ± 10	0.892
Male n (%)	3 (33.3%)	3 (30%)	0.737
Dialysis vintage (months)	93 (40; 140)	110 (58; 140)	0.509
Weight (kg)	58.7 ± 11	58.3 ± 7.8	0.934
Body mass index (kg/m^2^)	22.9 ± 3.6	23.8 ± 1.7	0.500
ESS (score)	8 (3; 12)	7 (4; 11)	0.853
Pain (n)			0.034
*Mild (0–2)*	4	0	
*Moderate (3–7)*	3	3	
*Severe (7–10)*	2	7	
Pruritus (n)			0.809
*Mild (0–2)*	5	5	
*Moderate (3–7)*	4	5	
*Severe (7–10)*	0	0	
Sleeping pills use, n (%)	2	3	0.701
Erythropoietin (UI/kg/week)	183 (59, 287)	197 (166, 284)	0.549
iPTH (pg/ml)	1,554 (854; 2,544)	1,579 (1131; 2,844)	0.645
Alkaline phosphatase (U/L)	283 (184; 1,359)	466 (268; 996)	0.702
Serum phosphate (mg/dl)	4.50 ± 0.85	6.1 ± 1,25	0.005
Serum calcium (mg/dl)	9.7 ± 0.75	9.8 ± 0,83	0.964
25 (OH)-vitamin D (ng/ml)	25.9 ± 9	23.1 ± 8,5	0.497
Hemoglobin (g/dl)	12.7 ± 1.14	11.9 ± 2,2	0.328
Serum Ferritin (ng/ml)	315 (240; 465)	359 (107; 596)	0.857
Iron (μg/ml)	58(36; 67)	57 (42; 72)	0.679
Albumin (g/dl)	4,3 ± 0,31	4,1 ± 0,72	0.515
Fetuin-A (ng/ml)	3172 (2946; 3964)	3358 (3028; 3663)	0.842
PLMI	3 (0; 12)	25 (2; 75)	0.063
PLMI >15 (events/hr)	3	5	0.650

Values are presented as mean ± SD or median (25th, 75th percentile), unless indicated otherwise.

ESS, Epworth sleepiness scale; iPTH, intact parathyroid hormone; PLMI, periodic leg movement index; AHI, apnea and hypopnea index.

**Fig 1:**
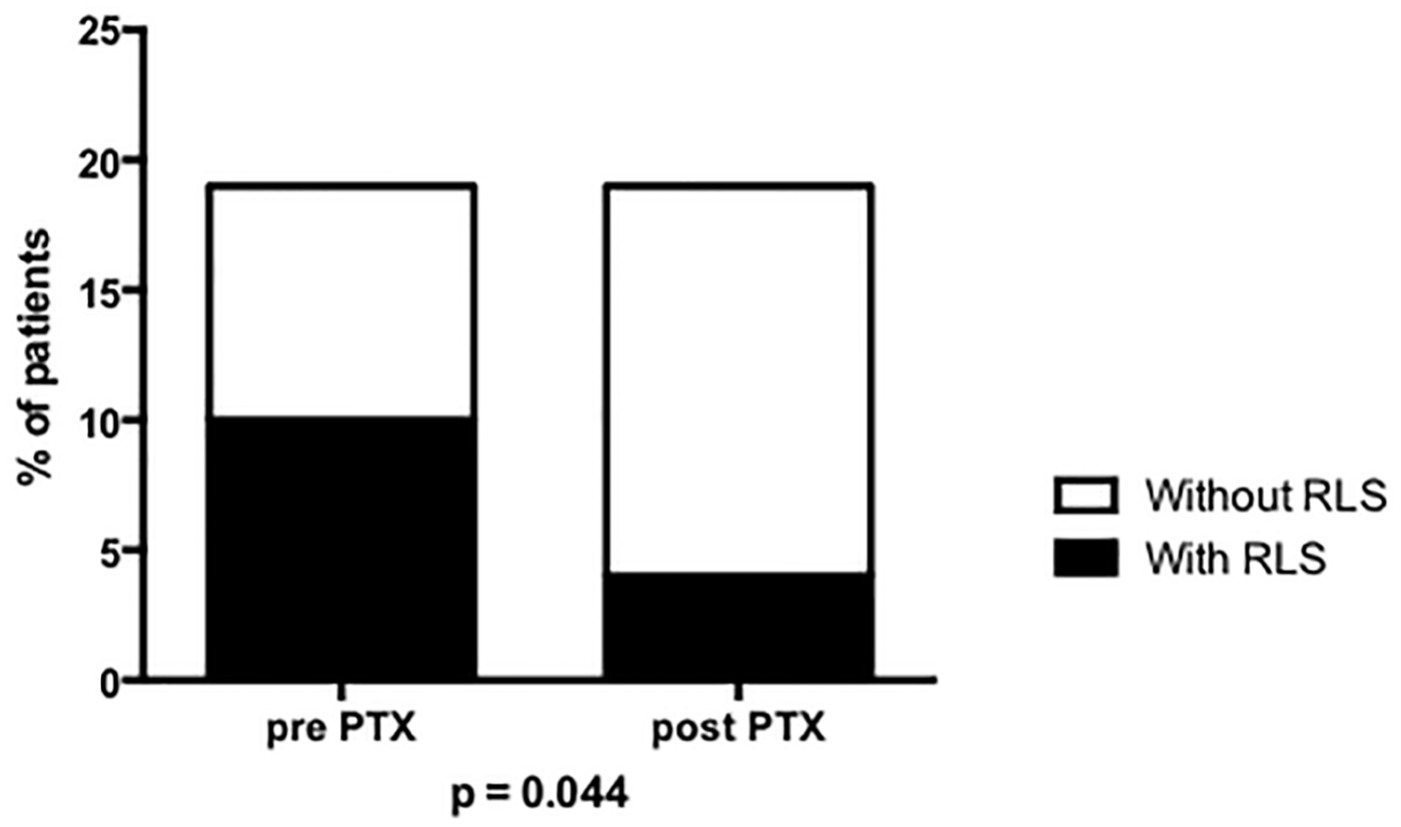
Prevalence of restless leg syndrome (RLS), pre- and post-Figure 1. Pparathyroidectomy.

**Fig 2:**
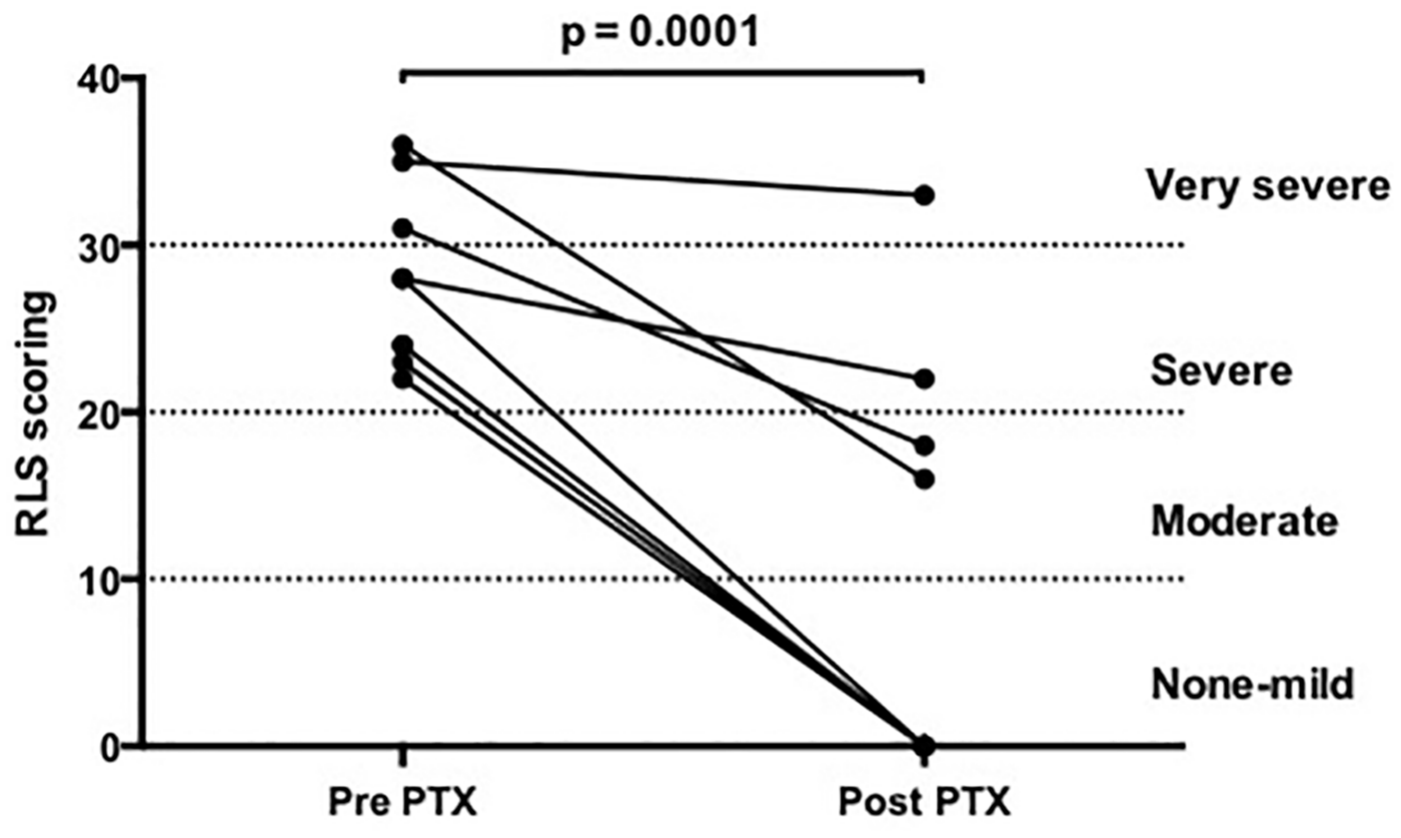
Scoring obtained with the international restless leg syndrome (RLS) severity scale questionnaire, pre- and post-parathyroidectomy.

As shown in Table 2, from pre- to post-PTX, there were no changes in weight, body mass index and neck circumference. There was an improvement in RLS after surgery: RLS decreased from 52.6% to 21% from pre- to post-PTX (p = 0.044) (Fig 1). In addition, the percentage of those suffering from severe to very severe RLS (IRLS score > 20) decreased from 90% to 20% in these patients (p = 0.017; Fig 2). As expected, there was an alleviation of pain and pruritus, accompanied by a reduction of serum PTH, phosphate, calcium and alkaline phosphatase. No significant differences were shown in hemoglobin, ferritin, iron and serum albumin levels between pre- and post-PTX. Serum levels of 25(OH)-vitamin D increased after PTX due to supplementation to all patients irrespective of the presence of RLS. Fetuin-A also increased following PTX.

**Table 2:** Demographic, clinical and biochemical characteristics pre- and post-parathyroidectomy (PTX).

Variable	Pre-PTX	Post-PTX	p
Weight (kg)	58.5 ± 9.2	59.4 ± 9.2	0.254
Body mass index (kg/m2)	23.4 ± 2.7	23.8 ± 3.3	0.225
Neck circumference (cm)	36.4 ± 2.9	36.7 ± 3.0	0.157
Pain, n (%)			0.025
Mild (0–2)	4 (21.0)	13 (68.4)	
Moderate (3–7)	6 (31.6)	6 (31.6)	
Severe (7–10)	9 (47.4)	0	
Pruritus, n (%)			0.033
*Mild (0–2)*	10 (52.6)	16 (84.2)	
*Moderate (3–7)*	9 (47.4)	2 (10.5)	
*Severe (7–10)*	0	1 (5.3)	
ESS score	7 (4, 10)	7 (5, 12)	0.495
ESS score > 10, n (%)	6 (31.6)	7 (36.8)	0.732
Sleeping pills use, n (%)	5 (26.3)	2 (10.5)	0.209
Erythropoietin (UI/kg/week)	195 (162, 267)	185 (113, 261)	0.376
Antihypertensive drugs, n (%)			0.376
*1 class*	5 (26.3)	7 (36.8)	
*2 classes*	4 (21)	2 (10.5)	
*3 classes*	5 (26.3)	2 (10.5)	
Restless Leg Syndrome, n (%)	10 (52.6)	4 (21.0)	0.044
Restless Leg Syndrome severity scores	27.9 ± 5.2	9.9 ± 12.6	0.0003
iPTH (pg/mL)	1554 (972, 2814)	71 (40, 189)	< 0.0001
Alkaline phosphatase (U/L)	366 (260, 1357)	110 (71, 177)	< 0.0001
Serum total calcium (mg/dl)	9.7 ± 0.8	8.1 ± 1.4	< 0.0001
Serum ionic calcium (mg/dl)	5.0 ± 0.3	4.3 ± 0.7	< 0.0001
Serum phosphate (mg/dl)	5.3 ± 1.3	4.5 ± 1.6	0.015
25 (OH)-vitamin D (ng/ml)	24.4 ± 8.6	35.5 ± 8	0.0009
Hemoglobin (g/dl)	12.3 ± 1.8	11.9 ± 2.2	0.589
Ferritin (ng/ml)	337 (167, 548)	383 (268, 663)	0.641
Iron (μg/ml)	58 (41, 69)	62 (43, 84)	0.578
Serum Albumin (g/dl)	4.2 ± 0.5	4.4 ± 0.5	0.277
Fetuin-A (ng/ml)	3330 ± 464	3879 ± 635	0.042

Values are presented as mean ± SD or median (25th, 75th percentile), unless indicated otherwise.

ESS, Epworth sleepiness scale; iPTH, intact parathyroid hormone.

Polysomnography data pre and post PTX showed only a tendency toward to improvement of sleep efficiency (p = 0.088) while analysing the entire group. PTX had no effect on apnea-hypopnea index. When analysing only patients with RLS pre and post PTX (Table 3), we have confirmed all biochemical changes observed in the entire group. Sleep parameters such as sleep efficiency and latency, as well as the percentage of REM sleep have improved.

**Table 3:** Clinical, biochemical and polysomnographic findings pre- and post-parathyroidectomy (PTX) of patients with Restless Legs Syndrome (RLS).

Variable	Pre-PTX	Post-PTX	p
Weight (kg)	58.4 ± 8.4	60.1 ± 7.0	0.795
Body mass index (kg/m^2^)	24.0 ± 1.8	24.7 ± 3.1	0.592
ESS (score)	7 (5; 12)	9 (6; 13)	0.906
ESS score > 10, n (%)	3 (30)	4 (40)	1
Restless Leg Syndrome severity scores	27.9 ± 5.5	11.3 ± 2.9	0.008
iPTH (pg/mL)	1336 (1114, 2903)	70 (34, 100)	0.016
Alkaline phosphatase (U/L)	444 (262, 1243)	135 (100, 282)	0.008
Serum total calcium (mg/dl)	9.7 ± 0.8	7.7 ± 1.5	0.010
Serum phosphate (mg/dl)	5.9 ± 1.3	4.6 ± 1.8	0.049
25 (OH)-vitamin D (ng/ml)	23.6 ± 9.6	33.2 ± 8.6	0.103
Hemoglobin (g/dl)	11.4 ± 2.1	12.4 ± 2.3	0.396
Ferritin (ng/ml)	359 (106, 595)	294 (164, 635)	0.812
Iron (μg/ml)	57 (42, 72)	61 (38, 72)	0.989
Serum Albumin (g/dl)	3.7 ± 0.5	4.3 ± 0.6	0.514
Fetuin-A (ng/ml)	3324 ± 416	4275 ± 283	0.031
TST (min)	336 ± 28	368 ± 39	0.250
Sleep efficiency (%)	80 ± 8	86 ± 5	0.035
Sleep onset (min)	17.0 ± 7.8	9.2 ± 4.4	0.002
Arousals/h	14.2 ± 6.4	12.9 ± 6.3	0.524
N1 (%)	10 (6; 15)	8 (6; 14)	0.312
N2 (%)	47 (37; 56)	48 (45; 53)	0.742
N3 (%)	21 (15; 37)	18 (16; 24)	0.547
REM (%)	19 (13; 23)	21 (16, 22)	0.008
PLMI (events/hr of sleep)	18 (0.6; 37)	16 (0; 52)	0.687
AHI (events/hr of sleep)	14 (2; 21)	13 (3; 21)	0.383
Mean Oxygen Saturation (%)	94 (92; 96)	95 (94; 96)	0.250
Lowest Oxygen Saturation (%)	87 (84; 91)	88 (82; 89)	0.859

Values are presented as mean ± SD or median (25th, 75th percentile), unless indicated otherwise.

ESS, Epworth sleepiness scale; iPTH, intact parathyroid hormone; TST, total sleep time; REM, rapid eye movement; PLMI, periodic leg movement index; AHI, apnea and hypopnea index.

## Discussion

The major findings of this study were as following: 1. Using standard and recommended criteria for the diagnosis of RLS, we confirmed a high prevalence of this syndrome in patients with ESRD on hemodialysis; 2. Pain, instead of paresthesia, was the main reported complain in these patients; 3. High serum phosphate was independently associated with RLS; 4. There was an alleviation of RLS symptoms following PTX; 5. Sleep parameters have improved in patients with RLS after PTX.

In agreement of previous studies [3–6], we found a high prevalence of RLS (52.6%), in patients with ESRD, although such comparison is not trustful, as not all studies have applied the IRLSSG diagnostic criteria.

There are two currently theories involved in the pathogenesis of RLS, iron and dopamine metabolisms, suggesting a dysfunction of hypothalamic dopaminergic cells that are the source of spinal cord dopamine. In this regard, patients with ESRD, who usually have iron depletion, may be at high risk of RLS. In the present study, however, the alleviation of RLS post PTX cannot be imputed to iron stores, as there was no change in iron, transferrin saturation and ferritin from pre- to post-PTX. Interesting, the severity of RLS was correlated with the dose of erythropoietin/kg/week, which may be related to inflammation and resistance to the action of erythropoietin.

Patients with RLS do not usually report pain, and parethesia is the most usual complain. Even the RLS international guidelines do not call attention for this symptom. We can speculate that, at least for patients with severe hyperparathyroidism, pain, though explained as bone pain, may reflect, in fact, the presence of RLS. Although we have applied a specific questionnaire for RLS diagnosis, it must be stressed that some symptoms such as pruritus and pain are very common among patients on dialysis, and might be alleviated by PTX, regardless of RLS presence. We trust that the diagnosis of RLS was not influenced by the presence of these symptoms. Indeed, several patients presented pruritus and pain and did not fit the criteria for RLS diagnosis. Nevertheless, “pain” instead of any other symptom, was the main complain we have verified among patients with RLS on hemodialysis, and the guidelines do not even mention to this particularity.

High levels of phosphate before parathyroidectomy were independently associated to the presence of RLS, a finding that was previously verified by Takaki and col. in patients on hemodialysis [9]. Phosphate can be considered a uremic toxin [16] based on its biological effects, and also based on adverse effects of hyperphosphatemia. It’s already known that high levels of phosphate are deleterious to the cardiovascular system [17]. Perhaps, phosphate may configure as a uremic toxin also due to its effects on sleep disorders. Although already described, we found no association between RLS and PTH, possibly because of the study design where all patients have high levels pre- and low levels post-PTX, regardless of the RLS presence.

Hyperparathyroidism is almost universal in ESRD. PTX remains the primary therapeutic means for medication-refractory SHPT patients. Bone pain, pruritus, fractures, cardiovascular disease and mortality are its major outcomes and all of them can be reduced by such surgical treatment [18], with a significantly reduction in rates of all-cause and cardiovascular mortality in these patients [19]. We evaluated patients with severe HPTS [median serum levels of iPTH 1554 (972, 2814)] refractory to available medical therapy, including dietary phosphate restriction, calcium-free phosphate binders, and calcitriol. This situation has given us a unique opportunity to assess an alleviation of PTX-related RLS. We confirmed previous report [20], demonstrating that PTX could relieve RLS in patients with severe hyperparathyroidism. In addition, we have demonstrated that PTX could reduce the severity of RLS in those patients that remained with this diagnosis. An important aspect of our study was the use of the international and validated questionnaire for the RLS diagnosis, and the face-to-face interview, as other comorbidities can mimic the symptoms of RLS. Furthermore, another unique finding of the current study is showing that PSG data did improve after parathyroidectomy. Previous studies that have demonstrated an increase in sleep duration and improvement on sleep quality, either following parathyroidectomy [20,21], or when comparing patients with and without RLS, [21,22], were based on subjective data with no polysomnography measurement. We found no improvement on sleep respiratory parameters, which was not a surprise. However, we expected to find some improvement in sleep efficiency, which did not occur. Due the small number of patients evaluated, further studies with PSG data are necessary before definitely conclude whether PTX is capable to improve sleep quality.

Hyperparathyroidism is associated with increased risk of other sleep disorders, such as insomnia, accompanied by bone pain, pruritus and general weakness [23]. Previous studies have shown an improvement of sleep disorders, sleep hours per night and severity of insomnia following PTX, in association with amelioration of those symptoms [21]. However, there was no PSG data confirming these findings. In the present study, we have demonstrated that successful PTX relieved the symptoms of pain and pruritus, in association with an improvement on sleep architecture.

As an additional finding, we have confirmed that serum Fetuin-A levels increased after PTX, which was previously demonstrated by Wang and col [24]. Since Fetuin-A is one of the several potential vascular calcification inhibitors in chronic kidney disease patients [25], an increase in its levels may be beneficial, as it was associated with a significant reduction in the all-cause mortality of uremic patients [26]. Levels of 25(OH)-vitamin D increased after PTX as a result of oral supplementation, which could be contributed to the alleviation of RLS, as previously described [27]. However, as all patients receiving vitamin D supplementation, regardless the diagnosis of RLS, other studies are need to assess the role of vitamin supplementation in this population.

Certain limitations of this study should be considered. First, the relatively small sample size might have reduced the ability to find differences between patients with and without RLS. We partially overcome this issue, by analysing the same patient pre and post a major intervention (PTX). Also, it might influence the multivariable analyses, and the overfitting should not be completed eliminated. Another limitation is that we have tested the RLS in a short time period after PTX, and this result must be confirmed in a long-term study. Still, the straights of the present study are demonstrating, based on standard criteria, and face-to-face interview that PTX may improve RLS and sleep architecture.

In summary, RLS, a disable and painful condition in patients with ESRD can be relieved by parathyroidectomy. Serum phosphate is so far, the main marker of this disease. It is recognized that PTX can improve survival, and RLS should be also kept in mind as another potential target to indicate this surgical treatment.
